# Enhanced Antioxidant Activity of Fresh Fruits through Salicylic Acid/*β*-CD Hydroalcoholic Gels

**DOI:** 10.3390/gels8010061

**Published:** 2022-01-15

**Authors:** Zujin Yang, Youliang Guan, Hongbing Ji

**Affiliations:** 1 School of Chemical Engineering and Technology, Sun Yat-sen University, Zhuhai 519082, China; guanyliang@mail2.sysu.edu.cn; 2 School of Chemical Engineering, Guangdong University of Petrochemical Technology, Maoming 525000, China; 3 School of Chemistry, Sun Yat-sen University, Guangzhou 510275, China

**Keywords:** banana, decay, SA/*β*-CD inclusion complex, antioxidant activity, ONIOM

## Abstract

Oxidation is an important cause of fruit spoilage, and therefore improving the antioxidant capacity of fresh fruits is beneficial to their preservation. Herein, fresh-cut bananas were used as a type of fresh fruit and soaked in 75% hydroalcoholic gels containing salicylic acid (SA) or SA/*β*-CD inclusion complex (SA/*β*-CD). After treatment, they were placed in an atmosphere at 85% relative humidity at 20 °C for 12 days. A significant reduction in spoilage in bananas treated with the hydroalcoholic gels in the presence of SA/*β*-CD was observed, compared with those treated with gels in the presence or absence of SA. The free-radical-scavenging performances of SA and its complex were investigated using the DPPH (1,1-diphenyl-2-picryl-hydrazil) method. Based on the results, the significant increase in antioxidant activity was attributed to the fact that the inclusion complex could break the intramolecular hydrogen bonding of SA, thus efficiently eliminating ROS in the fruits. The formation of the inclusion complex was confirmed by experiments and theoretical calculations. Our findings indicate that treatment with SA/*β*-CD can provide an efficient method of maintaining postharvest quality and extending the shelf life of bananas.

## 1. Introduction

Banana (*Musa* spp.) is a type of fruit with important nutritional and commercial value, mainly grown in tropical and subtropical regions [1]. However, the short storage period of bananas has always been problematic. One of the main reasons for banana decay is reactive oxygen species (ROS) metabolism after harvest [2,3]. ROS such as superoxide anion radicals (O^2−^·), hydrogen peroxide (H_2_O_2_), hydroxyl radicals (OH·), and singlet oxygen are all by-products of aerobic metabolism [4]. Gill et al. confirmed that ROS could affect plant tissues by damaging nucleic acids, oxidizing proteins, and causing lipid peroxidation (LPO) [5]. Therefore, the removal of ROS using antioxidants could be an effective approach to extending the storage life of fresh fruits.

**Figure 1:**
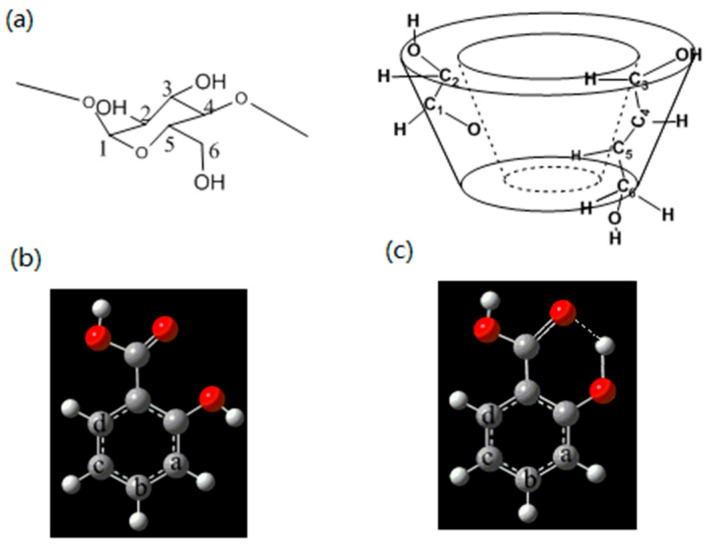
(**a**) Molecular structures of *β*-CD, with glucose unit connected via bonds 1–4; (**b**,**c**) molecular structures of SA.

Freezing is regarded as one of the most important methods of retaining fruit quality during long-term storage. Recently, much interest in food phenolic compounds has been aroused, due to their antioxidant properties and strong ability to remove free radicals. Salicylic acid (SA, Figure 1) is a natural phenolic component, mainly found in natural willow bark. It is considered to be one of the plant signal molecules used to regulate the growth of plants [6]. Previous studies have shown that SA could reduce fruit spoilage by increasing the elimination of reactive oxygen species in fruits [7,8]. Huang et al. indicated that pretreatment with SA and lower storage temperatures prolonged the postharvest life of navel oranges [9]. However, the adjacent carboxyl and hydroxyl groups of the SA monomer can form intramolecular hydrogen bonds, which might decrease its antioxidant activity towards fresh fruits [10]. 

One of the known methods of preventing the formation of intramolecular hydrogen bonding is to construct a host–guest complex between the SA and the host, e.g., cyclodextrins (CDs). CDs is a general term for different cyclic oligosaccharides. They are prepared from amylose with the help of cyclodextrin glucosyltransferase, synthesized by Bacillus [11,12,13]. The unique feature of CDs is that they can form inclusion complexes via non-covalent interactions in solution or in the solid phase. They are considered to be the simplest encapsulating systems. It has been reported that the antioxidant activity of phenolic components was improved after inclusion with CDs [14]. As the most accessible, the cheapest, and the most useful of the CDs, *β*-CD (Figure 1) is a potential candidate for use in the pharmaceutical, cosmetics, and food industries [15,16]. 

The purpose of this study was to explore the effect of SA/*β*-CD on controlling the decay of fresh fruits. In addition, the antioxidant activity of SA/*β*-CD was evaluated using a DPPH free-radical-scavenging test. The inhibition mechanism is also discussed with the help of experiments and theoretical calculations.

## 2. Results and Discussion

### 2.1. LC and EE of SA/β-CD Inclusion Complex

*β*-CD and some drug molecules that do not have biocompatibility have been prepared as inclusion compounds, which can increase the biocompatibility of the drugs and play a role in their slow release [17,18]. Different strategies for forming inclusion complexes, including co-precipitation, grinding, freeze-drying, and spray drying have been tested successfully. Vacuum drying is used to reduce the decomposition and volatilization of active ingredients. In this study, the loading capacity (LC) and the encapsulation efficiency (EE) for SA/*β*-CD (1:1 molar ratio) were 10.00% and 92.24%, respectively. 

### 2.2. Banana Decay Test

**Figure 2:**
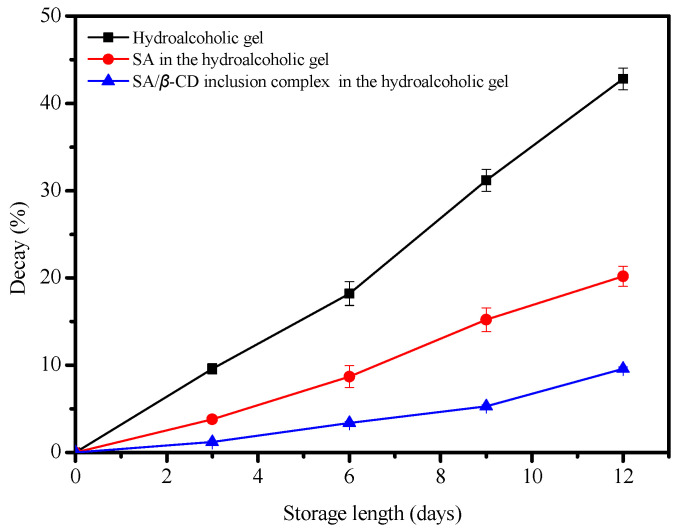
Effects of control, SA, and SA/*β*-CD treatments on decay rates of fresh-cut bananas during 12 days of storage at 85% RH at 20 °C.

A banana decay test was performed, and the results are shown in Figure 2. Bananas were soaked in hydroalcoholic gels containing SA/*β*-CD and SA, and a control sample with the gel only was also used. They were stored at 85% RH at 20 °C for 12 days. The results showed that the decay rate of the control was 42.8%, but a decay rate of only 20.2% was observed in the presence of SA. The most exciting result was the decay rate of only 9.6% for the bananas treated with SA/*β*-CD. This suggested that SA could decrease the decay rate of fresh-cut bananas by increasing the ability to eliminate reactive oxygen species in the fruits. However, the SA/*β*-CD-treated samples could considerably reduce the natural decay of the banana fruits. This could be due to the formation of intermolecular hydrogen bonds between SA and *β*-CD, which may enhance the antioxidation properties of the SA.

### 2.3. Antioxidant Activity

An assay of the DPPH free-radical-scavenging ability is usually applied for the analysis of the antioxidant activity of phenolic components [19]. The free-radical scavenger is paired with the DPPH single electron so that its absorption gradually disappears, and the rate of reduction is quantitatively related to the number of electrons it accepts. Therefore, it can be used for rapid quantitative analysis with a microplate reader [20]. Hence, we can evaluate the antioxidant capacity of SA by measuring the absorbance of DPPH radicals at 517 nm.

Table 1 shows the antioxidant properties of SA and SA/*β*-CD. The elimination ability of the DPPH radical was improved from 7.33% to 27.83% after SA was microencapsulated with *β*-CD, which means that the antioxidant activity of SA/*β*-CD increased approximately 3.8-fold over that of SA. This enhancement was ascribed to intermolecular hydrogen bonds between SA and *β*-CD, eliminating the intramolecular hydrogen bonding of SA and leading to the increased antioxidant capacity. The improvement in antioxidant activity via the formation of CD inclusion complexes with the phenolic compounds has previously been reported [21].

### 2.4. FTIR Analysis

**Figure 3:**
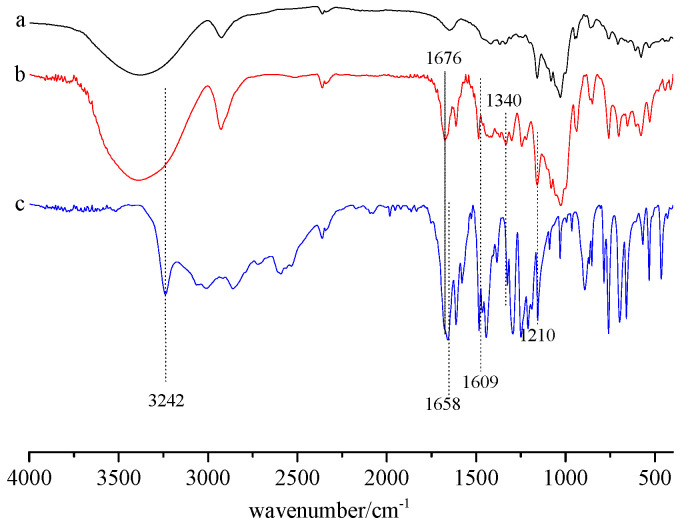
FTIR spectra of (**a**) *β*-CD, (**b**) SA/*β*-CD, and (**c**) SA.

FTIR is a very suitable and efficient tool which can determine the formation of inclusion complexes by measuring the shape, position, and intensity of characteristic peaks [22,23]. FTIR spectra of *β*-CD (a), SA/*β*-CD (b), and SA (c) are presented in Figure 3 from 400 to 4000 cm^−1^.

It was found that the prominent characteristic peaks of SA/*β*-CD (b) were consistent with those of *β*-CD (a), demonstrating that the overall structure of the complex was not changed. The characteristic bands of SA (c) were not observed in the spectrum of SA/*β*-CD, which could be due to overlapping by *β*-CD bands [24]. However, the characteristic bands of SA showed some obvious changes: (1) the band at 1609 cm^−1^ corresponding to C=C groups disappeared; (2) the intensity of the band at 1658 cm^−1^ due to C=O groups was observed to decrease and shift to 1676 cm^−1^ after the inclusion, which is in accordance with reported results [12,13,14]; (3) the intensity of the characteristic peak at 1210 cm^−1^ (C-O-H) was reduced in the spectrum of SA/*β*-CD; (4) the absorption band at 3242 cm^−1^ due to C-H in the benzene ring of SA disappeared in the IR spectrum of SA/*β*-CD. Therefore, SA was enclosed in the cavity of *β*-CD. This was also confirmed using 2D ROESY and theoretical studies, as discussed in the next section.

### 2.5. UV–Vis Analysis 

With the assumption of a 1:1 stoichiometry, the inclusion of SA (H) and *β*-CD (G) can be calculated from Equation (1).

(1) $$H+G\overset{K}{⇄}H-G$$

Under the conditions used, the concentration of *β*-CD was higher than that of SA, that is, [G]_0_ ≫ [H]_0_. Therefore, the binding constant of the complex formed was calculated using Equation (2), according to the Hildebrand–Benesi equation [25]:

(2) $$\frac{1}{A-A_{0}}C=\frac{1}{a}+\frac{1}{aK{\left(\beta -CD\right)}_{0}}$$

where *A* and *A*_0_ are the absorbances of SA in the presence and absence of *β*-CD, respectively. *K* is the stability constant of the complex when SA:*β*-CD is a 1:1 molar ratio, a is a parameter correlated with the variation in the molar absorption coefficients, and [*β*-CD]_0_ is the initial concentration of *β*-CD. 

**Figure 4:**
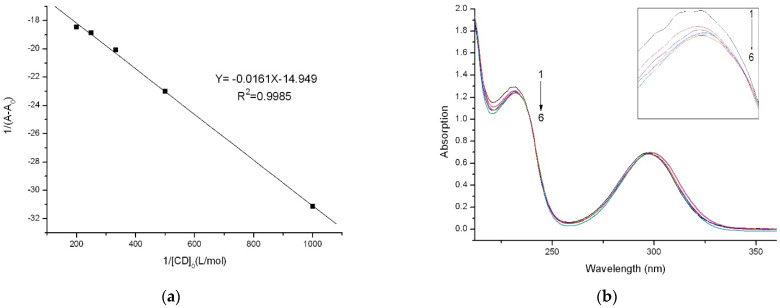
(**a**) The absorption spectra of 2.0 × 10^−4^ mol/L SA for different *β*-CD concentrations (×10^−3^ mol/L): (1) 0.0, (2) 1, (3) 2, (4) 3, (5) 4, and (6) 5, at 298 K; (**b**) Benesi–Hildebrand plot of 1/(A − A_0_) vs. 1/[*β* − CD]_0_ for SA.

For a given concentration of *β*-CD, *K* was calculated and is displayed in Figure 4b. Based on the good straight trend line, it can be seen that *β*-CD and SA can form a complex with a molar ratio of 1:1. Previous results implied that the *K*-value of the complex reflected the intensity of the weak interaction between host and guest, as expressed in Equation (2). From Figure 4, *K* for SA/*β*-CD was 928 M^−1^, which indicated that a stable complex was formed through the synergistic effect of the weak interactions between SA and *β*-CD. 

### 2.6. TGA Analysis

**Figure 5:**
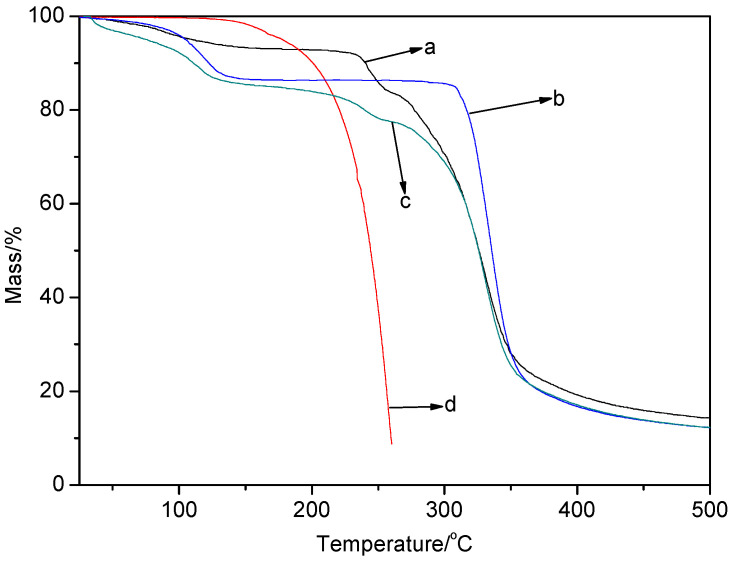
TG curves of: (**a**) SA/*β*-CD complex, (**b**) *β*-CD, (**c**) physical mixture of SA and *β*-CD, and (**d**) SA.

Thermogravimetric analysis (TGA) is an efficient method used to investigate the physical and chemical properties of complex formation. Here, TGA was performed in a N_2_ atmosphere, with a heating rate of 10 °C/min from 30 to 500 °C. The TG curves of SA/*β*-CD (a), *β*-CD (b), the mixture of SA and *β*-CD (c), and SA (d) are shown in Figure 5. 

As shown in Figure 5, the mass of SA was reduced by nearly 100% before 300 °C, which was ascribed to the volatility of SA. The TGA curve of *β*-CD presented two stages of weight loss. The first stage, up to about 110 °C with 11.5% weight loss, was due to the evaporation of water adsorbed by the *β*-CD. However, the physical mixture of SA and *β*-CD had a higher weight loss of 13.8% and the complex of SA/*β*-CD showed a lower weight loss of 4.3% at about this temperature. This difference was attributed to the fact that when SA was included into the cavity of *β*-CD, the crystal water in the cavity of *β*-CD was replaced by SA to form a stable complex. The temperature of 110 °C can effectively remove the water absorbed on the surface of *β*-CD and free SA in the physical mixture, but it cannot eliminate the SA encapsulated in the complex. The second stage of *β*-CD weight loss, up to 330 °C with about 70% weight loss, was due to the decomposition of *β*-CD, and similar results were also observed for the mixture and the SA/*β*-CD complex. However, the TGA curve of the SA/*β*-CD complex displayed a slow decrease up to 300 °C, implying that the SA was well protected and had better stability than in the physical mixture. Therefore, the weight loss of SA was significantly decreased, which was attributed to the fact that the weak interactions between *β*-CD and SA prevented the volatilization of SA.

### 2.7. NMR Analysis

NMR has been used to detect the weak interaction between CD and guests and to elucidate the structure of CD-based complexes [26]. Generally, by analyzing the change (Δδ = δ*_β_*_-CD_ − δ_inclusion complex_) in the corresponding peaks between CDs and its complexes, we can judge whether there is interaction between CDs and guests. When a guest is included into the cavity of the CD, shift changes in the H3 and H5 protons of *β*-CD are observed, which are due to the shielding effect of the *β*-CD cavity and changes in the microenvironment and conformation. Chemical shifts in *β*-CD and its complex with SA observed in ^1^H NMR spectra were analyzed and are listed in Table 2. All the *β*-CD peaks were clearly observed to shift in the inclusion complex, demonstrating that SA was included inside the *β*-CD [27]. However, Table 2 also shows that there were no chemical shift changes for the H1, H2, and H4 signals before and after forming the complex, and downfield chemical shifts for H3 and H5 protons were detected rather than for the other protons of *β*-CD, demonstrating that SA was deeply encapsulated into the cavity of *β*-CD, and a stable complex with a 1:1 molar ratio was formed [28].

**Figure 6:**
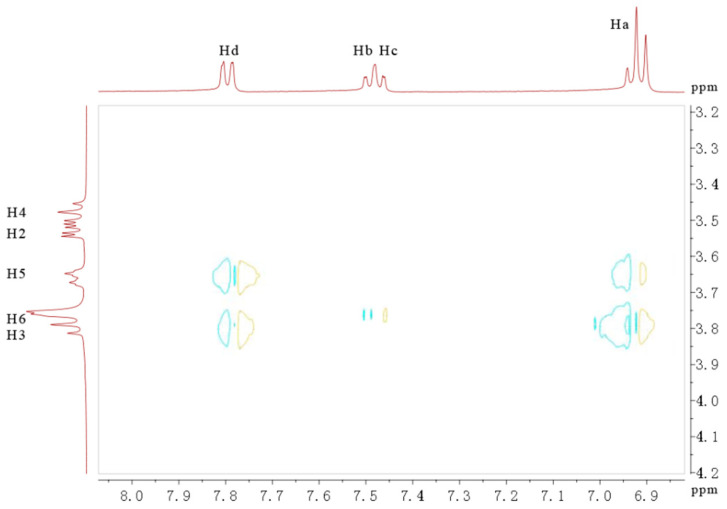
Part of the 2D ROESY spectrum of SA/*β*-CD in D_2_O.

By analyzing the chemical shift change for the H3 and H5 protons, the ^1^H NMR results confirmed the formation of the SA/*β*-CD complex. The 2D ROESY technique was further applied to investigate the interaction between SA and *β*-CD, since it can provide accurate conformational information. As shown in Figure 6, the 2D ROESY spectrum clearly showed that there were obvious cross peaks between the H3 and H5 protons of *β*-CD and the Ha and Hd of the aromatic ring group of the SA. Weak cross peaks between H6 and Hb and Hc of the SA protons were also observed. However, the cross peaks between Ha and Hd of the SA did not interact with the H3 and H5 external protons of *β*-CD. In addition, the interaction between the external H1, H2, and H4 protons of *β*-CD and SA protons could be ignored. The results indicated that a stable SA*/β*-CD inclusion complex had been formed, and the OH groups of the SA were located in the head-down direction in the *β*-CD.

### 2.8. Theoretical Investigation 

**Figure 7:**
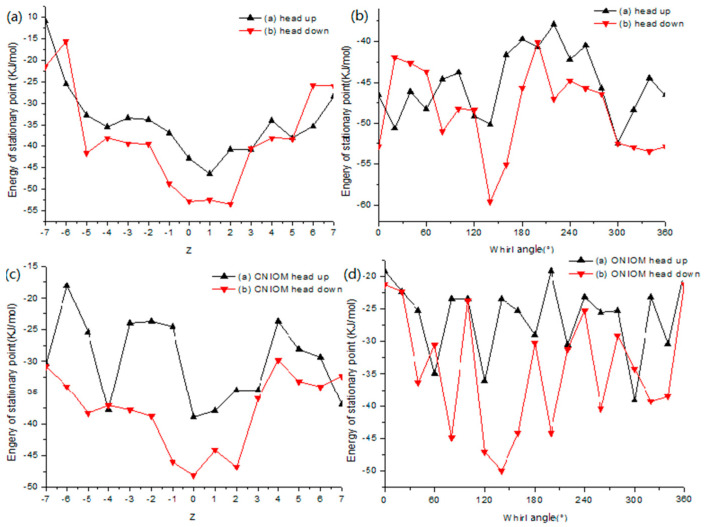
Binding energies between SA and *β*-CD calculated by PM3 method: (**a**) in different positions; (**b**) at different angles. The binding energies between SA and *β*-CD calculated by ONIOM (B3LYP/6-3lG:PM3) method: (**c**) in different positions; (**d**) at different angles.

The penetrations of SA into the cavities of *β*-CD were investigated using Gaussian 03, and the results are displayed in Figure 7. PM3 was used to optimize the geometries of the minimum-energy structures of the complexes in both the head-down and head-up orientations at 1 atm and 298.15 K in vacuo. Figure 7 shows the changes in binding energy in the inclusion process of SA into *β*-CD at different distances by changing the Z-coordinate and the *θ*-angle. The global minimum of the whole curve indicates that SA and *β*-CD can form a stable complex, because the binding energies of the inclusion complex calculated by PM3 were negative (Table 3). The binding energies of the most stable isomers in the pass through the cavity of *β*-CD were −52.53 kJ·mol^−1^ (head-up orientation) and −59.58 kJ·mol^−1^ (head-down orientation), respectively. The results calculated via PM3 indicated that the energy of the SA*/β*-CD complex in the head-down orientation was lower than that of the SA*/β*-CD complex in the head-up orientation, indicating that the SA*/β*-CD complex in the head-down orientation was more stable than in the head-up orientation [29]. 

To improve the computational accuracy, the semi-empirical hybrid ONIOM method was used to explore the SA/*β*-CD inclusion complex [29]. The binding energies calculated by B3LYP/6-31G and the binding energies for the most stable inclusion complex are shown in Table 4. 

Table 4 indicates that the ONIOM (B3LYP/6-31G(d)) results showed a similar tendency to the results of PM3. The *BE* energies from the ONIOM calculations for SA/*β*-CD in the head-up orientation and SA/*β*-CD in the head-down orientation were −39.10 and −50.03 kJ·mol^−1^, respectively, implying that the complex in the head-down orientation is much more stable than the complex in the head-up orientation.

### 2.9. Optimized Structures of the Inclusion Complex

**Figure 8:**
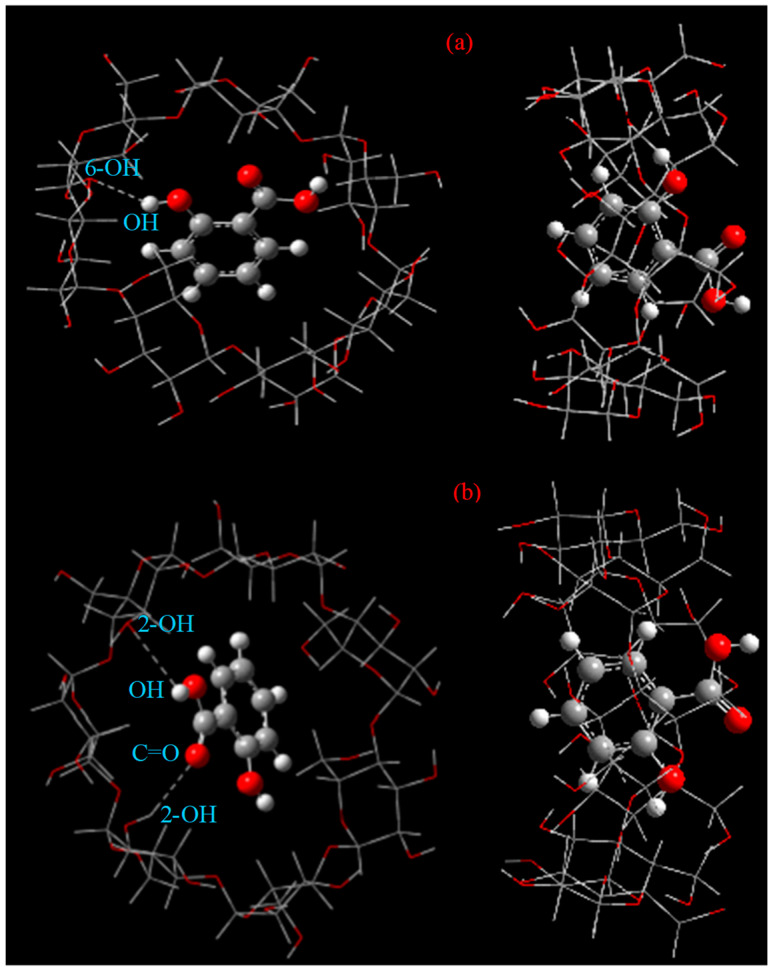
The structures of SA/*β*-CD with minimum energy achieved by ONIOM calculation: (**a**) passing process with head up and (**b**) with head down. Dotted lines are the H-bonds between SA and *β*-CD.

The ONIOM-optimized structures of the complex are shown in Figure 8 and Table 5. 

According to the reported data [30], the O⋅⋅⋅H distance in the O-H⋅⋅⋅O interaction is less than 3.2 Ǻ, and the angle at H is more than 90°. Furthermore, an O-H⋅⋅⋅O or C-H⋅⋅⋅O interaction often maintains a stable energy of 16–25 kJ·mol^−1^ or 0.7–2.8 kJ·mol^−1^ as the binding energy. Based on the ONIOM method, the optimized geometric configuration suggested that the nearest distances between the hydrogen atom of SA and the oxygen atom of the secondary hydroxyl group of *β*-CD were 2.549 Ǻ and 2.583 Ǻ for the head-up and head-down orientations, respectively, as listed in Table 5. The O-H⋅⋅⋅O bond angles were 147.272° and 118.587°, respectively. The SA molecule accepted an O-H⋅⋅⋅O bond from the 2-OH or 3-OH group located on the head down of the *β*-CD. This suggested that there was a hydrogen bond between β-CD and SA. As shown in Figure 8, the complex in the head-down orientation was more stable due to this interaction. Therefore, it is worth noting that hydrogen bond interactions play a vital role in SA/*β*-CD. This also explains the improved antioxidant capacity of the SA/*β*-CD complex.

## 3. Materials and Methods

### 3.1. Chemicals

*β*-CD was purchased from Shanghai Boao Biological Technology Co., Ltd., China. SA was supplied by Tianjin Yongda Chemical Reagent Co., Ltd., Tianjin, China. DPPH was obtained from Sigma-Aldrich, USA. All other reagents and solvents were of analytical grade. Deionized water was used throughout. 

### 3.2. Preparation of SA Inclusion Complex with β-CD

The inclusion complex of SA with *β*-CD (1:1 molar ratio) was prepared using the co-precipitation method. Briefly, 1.135 g of *β*-CD (1 mmol) was dissolved in 25 mL of water at 50 °C to form a clear solution. Then, 0.138 g of SA (1 mmol), dissolved in 5 mL of ethanol, was added to the *β*-CD solution. The mixture was kept at the same temperature for 2 h at 200 rpm. When a clear solution was formed, it was cooled to 4 °C for 24 h and then filtered using a 0.22 μm membrane filter, frozen, and freeze-dried for 24 h.

### 3.3. Content of SA in the Complex 

The content of SA in the complex was calculated based on the method in [31]. Different concentrations of SA (0.03, 0.06, 0.09, 0.12, 0.15, 0.18, 0.21, and 0.24 mmol/L) with anhydrous ethanol as the solvent were measured using a Hitachi UV-2450 spectrophotometer. The absorbance was adjusted to 235 nm. The relationship between the concentration of SA and the absorbance was determined. The SA in the inclusion complex was extracted and diluted with anhydrous ethanol for spectrophotometric measurements. The encapsulation efficiency (EE) and loading capacity (LC) of the complexes were obtained according to:

(3) $$\mathrm{EE}(\%)=\frac{(\mathrm{Total}\;\;\;\;\;\;\;\mathrm{SA}-\mathrm{Surface}\;\;\mathrm{SA})\;\mathrm{in}\;\mathrm{the}\;\mathrm{inclusion}\;\mathrm{complexes}}{\mathrm{Total}\;\;\mathrm{SA}}\times 100\%$$

(4) $$\mathrm{LC}(\%)=\frac{(\mathrm{Total}\;\;\;\;\;\;\;\mathrm{SA}-\mathrm{Surface}\;\;\mathrm{SA})\;\mathrm{in}\;\mathrm{the}\;\mathrm{inclusion}\;\mathrm{complexes}}{\mathrm{The}\;\mathrm{inclusion}\;\mathrm{complexes}}\times 100\%$$

### 3.4. Treatment of Banana Fruits and Their Decay Rates

Banana fruits were harvested from a banana plantation in Zhanjiang district, Guangdong. The fruits were selected for similar size, maturity, and appearance and divided into three groups. Banana fingers were dipped in 2 L of 75% hydroalcoholic gel (group 1, control group), SA (group 2, 500 μmol/L), or SA/*β*-CD (group 3, SA, 500 μmol/L) under laboratory conditions for 4 h with occasional shaking. After draining, the banana fingers were placed in moist chambers at 20 °C with a humidity of 85%. Samples were taken initially and after 3, 6, 9, and 12 days of storage, for analysis. 

Decay rates (DR) were recorded according to Equation (5).

(5) $$DR=\frac{DW}{IW}\times 100$$

Here, *DW* is the weight of spoiled fruits and *IW* is the initial weight of the fresh fruits. 

### 3.5. Antioxidant Activity of SA and Its Inclusion Complex

The ability of SA to scavenge DPPH was assessed according to methods previously reported in the literature [32,33]. A total of 2.716 mg of SA or 27.16 mg of SA/*β*-CD was dissolved in 3.5 mL of water. To evaluate their antioxidant activity, 1 mL of SA or SA/*β*-CD solution was added into 2 mL of ethanol solution containing 0.01 mg/mL of DPPH·. After the mixture was incubated for 90 min at 25 °C in the dark, the free-radical-scavenging activity could be calculated by monitoring the absorbance of the DPPH· solution at 517 nm in the presence of the SA/*β*-CD (or SA) and comparing it with that of the blank sample, which was a mixture of 1 mL of distilled water with 2 mL of DPPH·–ethanol solution. The percentage scavenging activity was calculated using Equation (6):

(6) $$\mathrm{Scavenging}\;\mathrm{activity}/\%=\frac{{(A}_{\mathrm{DPPH}}{-A}_{\mathrm{Sample}})}{A_{\mathrm{DPPH}}}\times 100\%$$

where A_DPPH_ and A_Sample_ are the absorbances of the DPPH solutions in the absence and presence of SA or SA/*β*-CD complex, respectively. All reported data were repeated three times.

### 3.6. Determination of Stability Constant

The stability constant of *β*-CD with SA was calculated using UV–vis spectrophotometry (UV-2450, Shimadzu, Japan) at 235 nm. Different concentrations of *β*-CD (0, 1.0, 2.0, 3.0, 4.0, and 5 × 10^−3^ mol/L) were mixed with 2.0 × 10^−4^ mol/L SA. All the solutions were stirred at 200 rpm and kept at 25 °C for 6 h before the measurements.

### 3.7. Characterization of the Inclusion Complex

The Fourier transform infrared (FTIR) spectra of the samples were collected on a Nicolet 6700 spectrometer (Thermo Scientific, USA) with KBr pellets, in the region 4000—400 cm^−1^. The 1H NMR and 2D ROESY spectra were acquired using a Bruker 400 spectrometer (Bruker BioSpin AG, Fallanden, Switzerland) at room temperature. D_2_O was used as an internal reference [34]. The TG experiments were performed using a NETZSCH STA 449 C thermal analysis system. All the samples were about 6.0 mg and were heated to 550 °C at a rate of 10 °C/min under a constant N_2_ flow.

### 3.8. Computational Methods

**Figure 9:**
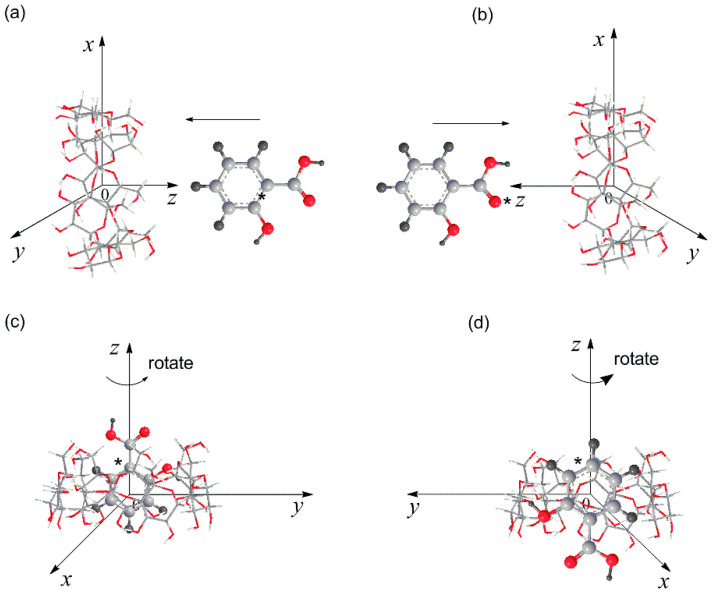
Coordinate systems used to define the complex processes: passing process for (**a**) head-up and (**b**) head-down orientations; circling process of (**c**) head-up and (**d**) head-down orientations. The labeled carbon atom (C*) of the guest molecule is used to express the relative position between the guest and host when the guest passes through the cavity of the *β*-CD.

All theoretical calculations were carried out using the Gaussian 03 software package [35,36]. *β*-CD and SA were the host molecule and the guest model, respectively. The geometry of *β*-CD was built using CS Chem3D Ultra (version 8.0) and was fully optimized using the PM3 method without imposing any symmetry restrictions [37]. The glycosidic oxygen atoms of *β*-CD were placed on the X–Y plane, and their center was defined as the center of the Cartesian coordinate system. The primary hydroxyl groups of *β*-CD were pointed toward the positive direction of the *Z*-axis. The inclusion complex was constructed from the PM3-optimized *β*-CD and the corresponding guest molecule. The longer dimension of the guest molecule was initially placed onto the *Z*-axis, and the relative position between the host and the guest was measured as the Z-coordinate of the labeled carbon atom of the guest, as shown in Figure 9 [38]. The coordinate systems for the inclusion process of the guest into *β*-CD are shown in Figure 9. The inclusion complex was emulated by introducing the guest molecule at one end of the *β*-CD and allowing it to pass through the *β*-CD in steps. For each step, the geometry of the complex was fully optimized by PM3 without imposing any symmetry restrictions. DFT single-point calculations at the level of B3LYP/3-21G* were performed for the PM3-optimized complex in vacuo. The binding energy (*BE*) could be expressed as in Equation (7):

*BE* = *E_complex_* − *E_host_* − *E_guest_* (7)

In this equation, *E_complex_* is the total energy of the inclusion complex, *E_guest_* is the total energy of the guest, and *E_host_* is the total energy of the host.

## 4. Conclusions

In this study, an SA/*β*-CD complex was successfully prepared. Compared to the hydroalcoholic gel or SA, the decay rate of banana fruits was significantly decreased after treatment with SA/*β*-CD hydroalcoholic gel. The antioxidant activity of SA was improved when it was included by *β*-CD in the hydroalcoholic gel. The complex was further analyzed by FTIR, UV–Vis, TG, ^1^H NMR, and 2D NOSEY. The complexation process of SA with *β*-CD was also determined using the two-layered hybrid ONIOM method. A 1:1 SA/*β*-CD complex with a stability constant of 928 M^−1^ was determined. The TGA results also indicated that the thermal stability of SA was significantly enhanced due to the formation of the inclusion complex. ^1^H NMR and 2D NOSEY studies confirmed the inclusion and provided information on the geometry of SA inside the *β*-CD cavity. Theoretical calculations and the NMR results explained why the stability and the antioxidant activity were increased when SA was included into *β*-CD. In brief, the SA/*β*-CD complex could be used to counter oxidation and therefore preserve fruit.

## Figures and Tables

**Table 1:** Antioxidant activity of SA and SA/*β*-CD.

Samples	DPPH-Scavenging Activity (%)
SA	7.33 ± 0.52
SA/*β*-CD	27.83 ± 0.74

**Table 2:** 2D ROESY cross peaks (present (+) or absent (-)) and ^1^H NMR chemical shift of *β*-CD, the inclusion complex of *β*-CD, and SA in D_2_O.

	H1	H2	H3	H4	H5	H6
δ*_β_*_-CD_ (ppm)	5.123	3.751	4.021	3.640	3.911	3.934
δ_inclusion_ (ppm)	5.102	3.684	3.900	3.637	3.788	3.923
Δδ (ppm) ^a^	0.001	0.006	0.121	0.003	0.123	0.011
Ha (~6.92 ppm)	-	-	+	-	+	+
Hb (~7.50 ppm)	-	-	-	-	-	+
Hc (~7.46 ppm)	-	-	-	-	-	+
Hd (~7.78 ppm)	-	-	+	-	+	+

^a^ Δδ = δ*_β_*_-CD_ − δ_inclusion complex._

**Table 3:** Binding energies for SA/*β*-CD calculated by PM3 method.

	*BE*/(KJ·mol^−1^)	*Z*_C*_/Å	*θ*/°
Head up	−52.53	1	300
Head down	−59.58	0	140

**Table 4:** Binding energies for SA/*β*-CD calculated by ONIOM (B3LYP/6-31G: PM3) method.

	*BE*/(KJ·mol^−1^)	*Z*_C*_/Å	*θ*/°
Head up	−39.10	1	300
Head down	−50.03	0	140

**Table 5:** Type, bond length (R), and bond angle (A) obtained by ONIOM (B3LYP/6-31G: PM3).

SA/*β*-CD	Type	R (Å)	A (°)	*θ*/°
head up	O58-H136···O (156)	2.549	147.272	300
head down	O157-H163···O (62)	2.583	118.587	140

## Data Availability

Not applicable.
